# Function of low ADARB1 expression in lung adenocarcinoma

**DOI:** 10.1371/journal.pone.0222298

**Published:** 2019-09-06

**Authors:** Xiang Wang, Zhijie Xu, Xinxin Ren, Xi Chen, Jie Wei, Wei Lin, Zhi Li, Chunlin Ou, Zhicheng Gong, Yuanliang Yan

**Affiliations:** 1 Department of Pharmacy, Xiangya Hospital, Central South University, Changsha, Hunan, China; 2 National Clinical Research Center for Geriatric Disorders, Xiangya Hospital, Central South University, Changsha, Hunan, China; 3 Department of Pathology, Xiangya Hospital, Central South University, Changsha, Hunan, China; 4 Center for Molecular Medicine, Xiangya Hospital, Key Laboratory of Molecular Radiation Oncology of Hunan Province, Central South University, Changsha, China; West Virginia University, UNITED STATES

## Abstract

Adenosine deaminase RNA-specific B1 (ADARB1), an adenosine-to-inosine (A-to-I) RNA-editing enzyme, has been found to play an essential role in the development of cancer. However, the specific function of ADARB1 in lung cancer, especially in lung adenocarcinoma (LUAD), is still not fully understood and requires further study. In our study, integrative bioinformatics were used to analyze the detailed function of ADARB1 in LUAD. By conducting bioinformatics analyses of several public databases, such as Gene Expression Profiling Interactive Analysis (GEPIA), GE-mini, and Oncomine, we found significantly decreased ADARB1 expression in LUAD cells and tissues. Moreover, RT-PCR and Western blot showed lower ADARB1 expression in H358 and A549 LUAD cells compared to human bronchial epithelial Beas-2B cells. Wound Healing Assay indicated that knockdown ADARB1 could promote LUAD cell metastasis. By using the Kaplan-Meier Plotter tool, we found that downregulation of ADARB1 was related to shorter first progression (FP), overall survival time (OS) and post-progression survival time (PPS). The relevant clinical data acquired from the Wanderer database indicated that the expression and methylation values of ADARB1 were significantly associated with the clinical characteristics of LUAD. Using DNA methylation inhibitor, we found DNMT inhibitor 5-aza-2-deoxycytidine (5-azaD) could promote the expression of ADARB1 and reverse the inhibition effect of ADARB1 in migration. In addition, functional enrichment analysis of ADARB1-associated coexpression genes was further conducted. Our investigation demonstrated that low levels of ADARB1 were specifically found in LUAD, and this gene might be a potential target in the diagnostic and prognostic evaluation of LUAD patients.

## Introduction

Lung cancer, the most common cancer worldwide, is the leading cause of cancer mortality in men and women[1]. Lung adenocarcinoma (LUAD), the most frequent subtype of lung cancer, has increased both in incidence and mortality[2]. Though traditional treatments, such as radiotherapy, chemotherapy and radical surgery, have been used clinically, the prognosis is still poor with a 5-year survival rate below 15%[3, 4]. Currently, patients cannot receive timely treatment because there is no effective early diagnostic method[5]. Therefore, novel target molecules must be clarified to strengthen the early diagnosis and treatment of LUAD.

Adenosine deaminase RNA-specific B1 (ADARB1), also known as ADAR2, is an adenosine-to-inosine (A-to-I) RNA-editing enzyme[6]. At present, research advancement in the field has revealed the relationship between ADARB1 and cancer. A recent study has found that ADARB1 was positively associated with the editing level of SLC22A3, a metastasis suppressor in esophageal squamous cell carcinoma[7]. Through next-generation sequencing transcriptomics, Chan *et al*. demonstrated that ADARB1 played a tumor suppressive role in gastric cancer through its catalytic deaminase domains[8]. Moreover, Valles et al. discovered that the expression of ADARB1 was correlated with prognosis of LUAD patients[9]. However, few studies exist that examine the effects of ADARB1 on pathological processes of LUAD.

Considering the important results of our current findings, we therefore conducted bioinformatics analyses to observe the detailed function and mechanism of ADARB1 in human LUAD. The results showed that the expression of ADARB1 was observed to be downregulated both in LUAD cells and tissues. Clinical characteristics were clearly associated with both the expression and methylation value of ADARB1 in LUAD. In addition, Kaplan–Meier Plotter analysis indicated that low levels of ADARB1 in LUAD were correlated with shorter first progression (FP), overall survival time (OS) and post-progression survival time (PPS). Kyoto Encyclopedia of Genes and Genomes (KEGG) and Gene Ontology enrichment (GO) were also used to analyze the biological functions of ADARB1-associated coexpression genes.

## Materials and methods

### Data acquisition and reanalysis using different bioinformatics methods

The bioinformatics analysis of ADARB1 in LUAD was conducted through a variety of bioinformatics algorithmic tools (S1 Table). The data obtained were already normalized by bioinformatics databases themselves, such as GEPIA[10] and GE-mini[11].

The cancer microarray data-mining platform, Oncomine, was used to identify gene expression profiles in human cancer tissues and cells[12]. From the database, we selected five studies (Hou Lung[13], Landi Lung[14], Okayama Lung[15], Selamat Lung[16] and Su Lung[17]) to evaluate the expression profiles of ADARB1 in LUAD tissues. The Cancer Cell Line Encyclopedia (CCLE) project supplies public access to a detailed genetic and pharmacologic characterization of more than 1400 cell lines[18]. This database enabled us to acquire ADARB1 expression patterns on the cellular level in LUAD. Three other databases, UALCAN[19], Gene Expression Profiling Interactive Analysis (GEPIA) and GE-mini, were used to reanalyze the cancer transcriptome data. As it contains individual gene expression profiles, Gene Expression Omnibus (GEO) contributed to research on specific profiles of interest based on gene annotation or precomputed profile characteristics[20]. The databases mentioned above could clearly identify the expression of ADARB1 in LUAD cells and tissues.

Wanderer, an interactive viewer, reserves the data of gene expression and DNA methylation in human cancers[21]. The Kaplan-Meier Plotter can then assess the effect of genes on survival time in cancer patients[22]. Using these two databases, we evaluated the relationship between ADARB1 and clinical characteristics to survival. Eastern Cooperative Oncology Group (ECOG) score, a prognosis factor, was used to evaluate the condition of posttreatment patients. DiseaseMeth version 2.0, the human disease methylation database, analyzes the association between disease and gene methylation[23].

Using the cBioportal web tool[24], we chose an LUAD dataset containing 230 microarray-sequenced samples[25] to discover the coexpression genes of ADARB1 in LUAD. Then, a protein-protein interaction (PPI) network of these coexpression genes was completed by employing the STRING database[26] and Cytoscape software[27]. GO and KEGG[28] pathway analyses of these ADARB1 coexpression genes were conducted by the web tools of WebGestalt[29] and DAVID[30] bioinformatics resource, respectively.

### Cells and reagents

The human lung cancer Beas-2B, H358, A549, 95C and 95D cell lines were obtained from the Cancer Research Institute, Central South University, China. Glioma cells were maintained in Roswell Park Memorial Institute (RPMI)-1640 medium (Gibco, Invitrogen, Carlsbad, CA, USA) with 10% fetal bovine serum (FBS, Gibco) and 100 U/ml penicillin-streptomycin (Gibco) at 37 °C and 5% CO_2_. 5-Azacytidine (100 mg) was purchased from MedChemExpress (HY-10586) and its exposed concentration was 5μM. siRNA (siADARB1) and ADARB1 antibody were purchased from RIBOBIO (siRNAPack_1999) and Proteintech (22248-1-AP), respectively.

### RNA extraction and Reverse Transcription PCR (RT-PCR)

Total RNA was extracted employing TRIzol reagent (Invitrogen) according to the manufacturer’s instruction and reverse transcribed to cDNA using the PrimeScript^™^ RT reagent kit (Takara, 6210). The RT-PCR assay was conducted through iTaqTM Universal SYBR green Supermix (Bio-Rad, United States), with β-actin as the internal control. The forward and reverse primer sequences were used as follows: ADARB1: 5’-GTGAAGGAAAACCGCAATCTGG-3’ and 5’- CAGGAGTGTGTACTGCAAACC-3’; β-actin: 5’-CATGTACGTTGCTATCCAGGC-3’ and 5’-CTCCTTAATGTCACGCACGAT-3’. Relative expression levels were decided using the 2-^ΔΔCT^ method. All reactions were run three or more times.

### Western blot analysis

The antibodies used in western blot were shown as follows: ADARB1 (22248-1-AP, Proteintech), α-Tubulin (sc-69969, Santa Cruz). Each protein sample, forty micrograms, was isolated by 8% SDS-PAGE. Then, the samples were transferred to the surface of polyvinylidene fluoride membrane and probed with the appropriate primary antibodies (SA00001-1, Goat anti-mouse IgG (H+L) HRP conjugate, Proteintech; SA00001-2, Goat anti-rabbit IgG (H+L) HRP conjugate, Proteintech) afterwards. The protein bands were visualized by immobilon western chemiluminescent reagents (WBKLS0500, Millipore).

### Wound healing assay

The cells were inoculated into 6-well plates and cultured in complete medium (37 °C, 5% CO_2_) to at least 95% confluence before wounds were created. To measure the cell migration, a plastic 100 mL pipette tip was used to scrape cells in a monolayer to creating wounds. Then, washed them three times in PBS and incubated with FBS-free RPMI-1640 medium. Subsequently, cells were cultured in either medium with 0 or 12 μM evodiamine for 0, 72 h. At the end of the incubation period, phase-contrast microscopy was employed to photograph the wounded area and migration cells at the wounded area. Finally, the relative wound closure was counted using Image J software[31] (https://imagej.nih.gov/ij/).

### Statistical analyses

#### Statistical methods for data obtained from databases

The statistical tests were conducted by using SPSS 12.0 software (IBM Analytics). The results were shown as the mean ± SD. Student’s t test, one-way ANOVA, multivariable analysis and K independent samples test were performed when appropriate. Data and the Spearman rank correlation were analyzed by Graphpad Prism 5 software and OriginLab, respectively. P < 0.05 was considered to be statistically significant.

#### Statistical methods for data acquired from laboratory

The statistical tests were conducted by using SPSS 12.0 software (IBM Analytics). Student’s t test, one-way ANOVA (Kruskal-Wallis test) and chi-square tests were performed when appropriate. P < 0.05 was considered to be statistical significance.

## Results

### ADARB1 had low expression in LUAD tissues and cell lines

**Fig 1:**
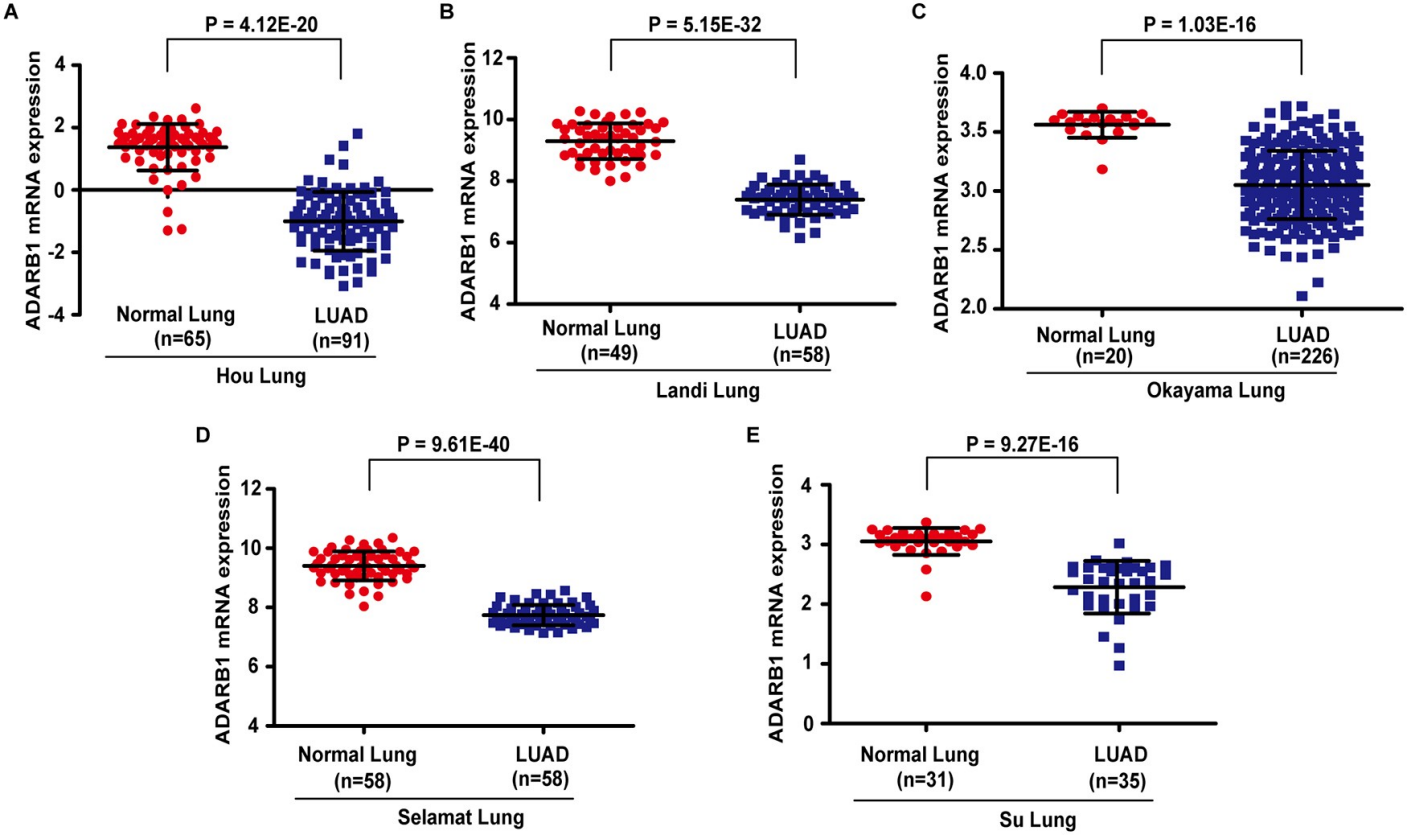
The Oncomine database indicated the downregulated ADARB1 in LUAD tissues. The expression of ADARB1 in five datasets (Hou Lung, Landi Lung, Okayama Lung, Selamat Lung and Su Lung) acquired from the Oncomine database.

**Fig 2:**
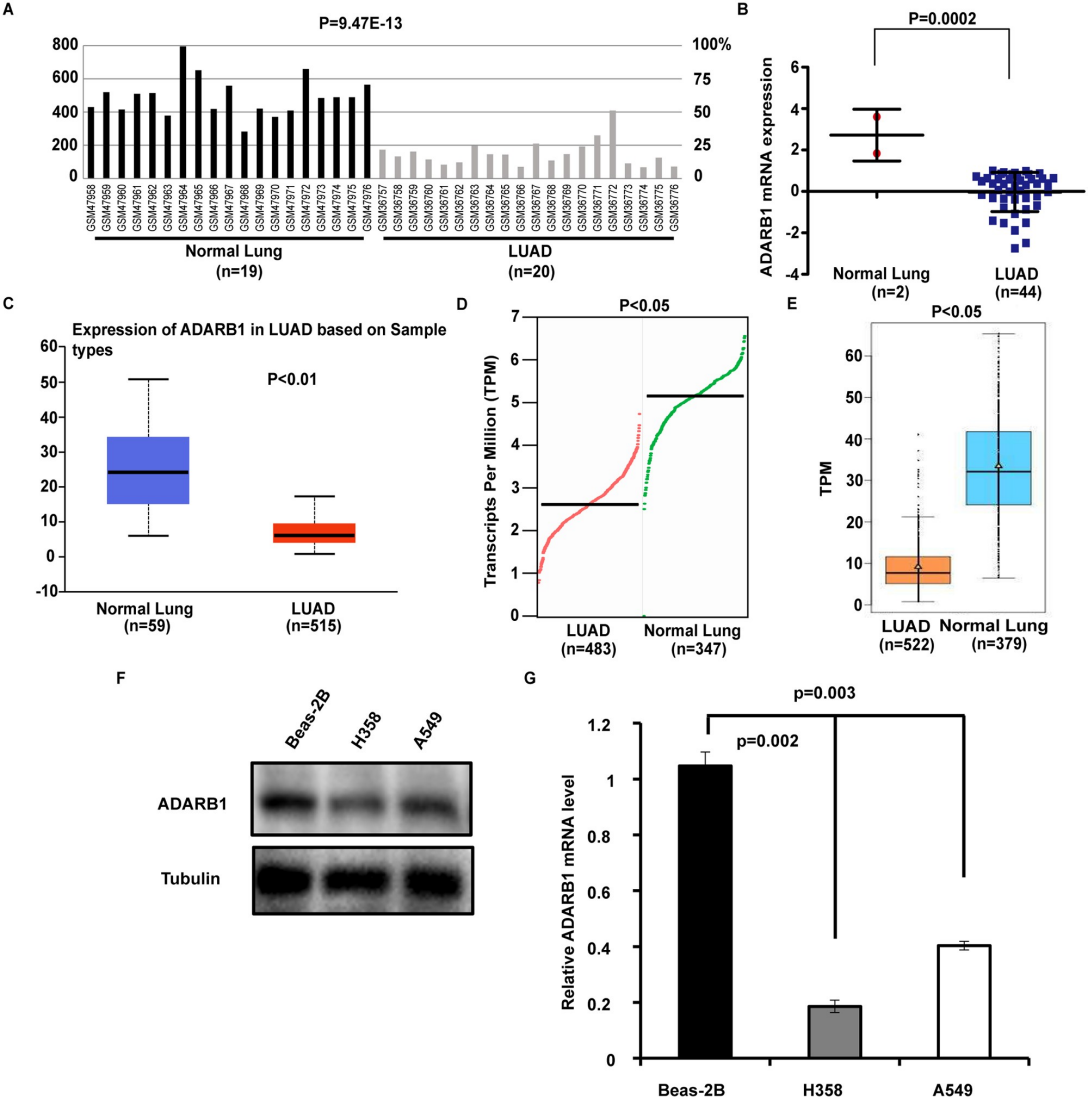
Analysis of ADARB1 expression levels in LUAD tissues and cell lines. (A) One dataset GSE2514, downloaded from the GEO database, described the mRNA expression of ADARB1 in LUAD in relation to normal lung tissues. (B) ADARB1 mRNA levels were significantly decreased in 44 LUAD cell lines, compared to 2 normal cell lines. (C-E) The mRNA expression of ADARB1 was evaluated from the database UALCAN, GEPIA and GE-mini, respectively. (F-G) Expression of ADARB1 was analyzed by qPCR and western blot.

To evaluate changes in ADARB1 expression in LUAD and adjacent non-tumor tissues, we analyzed the transcriptional levels of ADARB1 through seven independent bioinformatics databases. From the Oncomine database, we selected five microarray datasets, as shown in Fig 1(A)–1(E), and found that ADARB1 expression was significantly decreased in LUAD tissues. Dataset GSE2514[32] downloaded from the GEO database demonstrated that the expression of ADARB1 was downregulated in LUAD tissues (P = 9.47E-13) (Fig 2A). Through an exploration of the CCLE database, we discovered that ADARB1 expression was obviously reduced in 44 LUAD cell lines compared with normal lung cell lines (P = 0.0002) (Fig 2B). Similarly, using the UALCAN (Fig 2C), GEPIA (Fig 2D) and GE-mini (Fig 2E) databases, we further confirmed the downregulation of ADARB1 in LUAD tissues. As expected, we found that H358 and A549 displayed a lower level of ADARB1 compared to Beas-2B cell (Fig 2F and 2G). In conclusion, ADARB1 was significantly downregulated in LUAD tissues and cell lines, suggesting its anti-oncogenic role.

### ADARB1 could suppress LUAD cell metastasis

**Fig 3:**
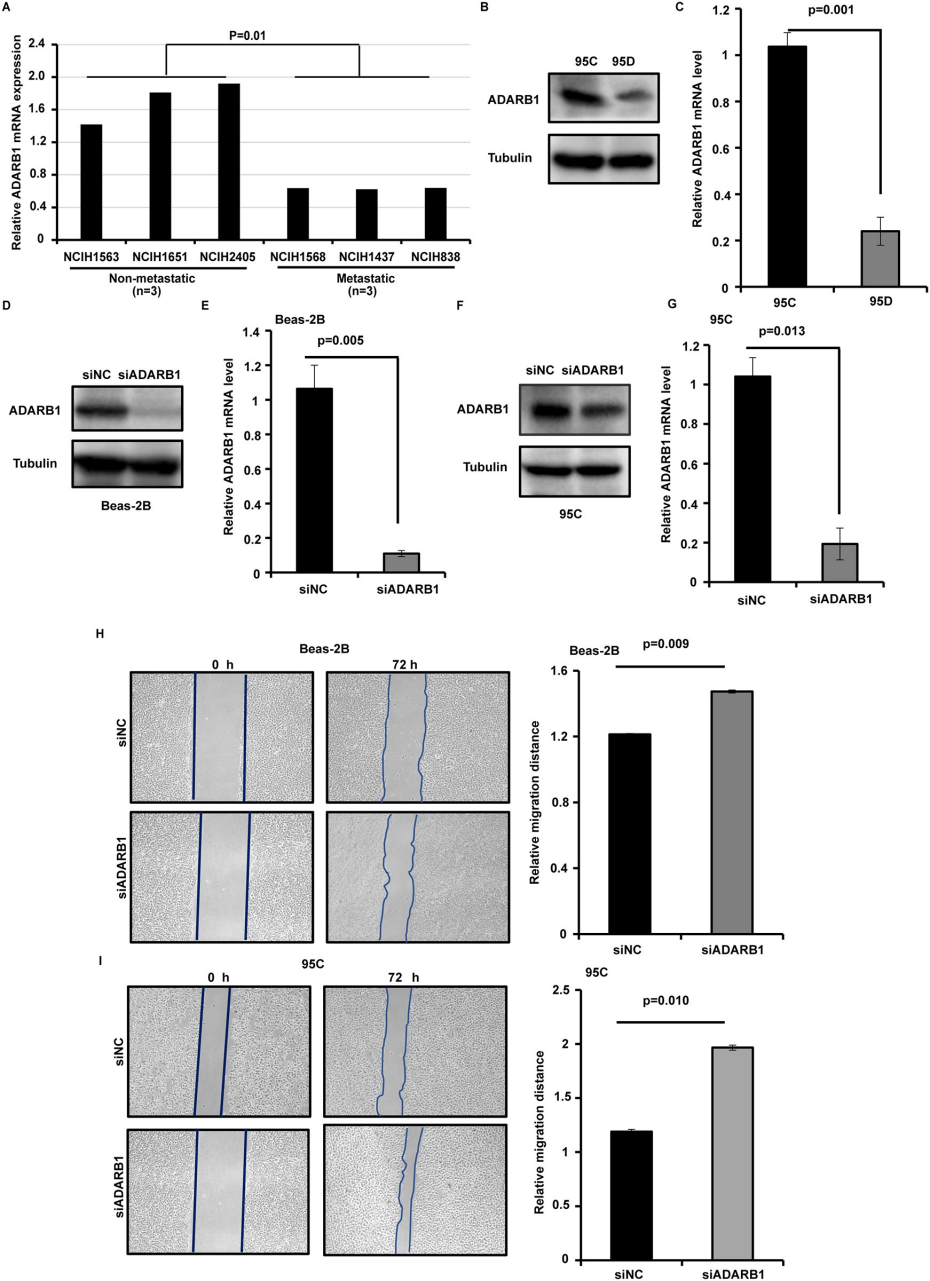
ADARB1 could suppress LUAD cell metastasis. (A) The CCLE database showed lower levels of ADARB1 in metastatic LUAD cells. Western blot (B) and RT-PCR (C) analyzed the expression of ADARB1 in 95C and 95D cells. The protein levels of ADARB1 after transfected with siADARB1 in Beas-2B (D) and 95C (F) cells. The mRNA levels of ADARB1 after transfected with siADARB1 in Beas-2B (E) and 95C (G) cells. The results of wound healing assay after transfected with siADARB1 in Beas-2B (H) and 95C (I) cells.

To further identify the function of ADARB1 in LUAD, the data from CCLE showed lower levels of ADARB1 in metastatic LUAD cells (NCIH1568, NCIH1437 and NCIH838) than in non-metastatic LUAD cells (NCIH1563, NCIH1651 and NCIH2405) (P = 0.01) (Fig 3A). We further investigated the expression of ADARB1 in high- (95D) and low-metastatic (95C) human lung cancer cells. Using RT-PCR and Western blot, ADARB1 were found obviously higher in 95C compared to 95D cells (Fig 3B and 3C). Additionally, we knocked down the expression of ADARB1 by siRNA in Beas-2B (Fig 3D and 3E) and 95C (Fig 3F and 3G) lung cancer cells and found that ADARB1 was significant lower in siADARB1 compared to siNC cells. The wound healing assay showed transfection with siADARB1 increased the metastasis ability compared to siNC in Beas-2B and 95C cells (P<0.01) (Fig 3H and 3I). This result suggests that ADARB1 could suppress LUAD metastasis and this hypothesis should be studied in further detail.

### ADARB1 expression was related to the clinical characteristics of LUAD patients

**Fig 4:**
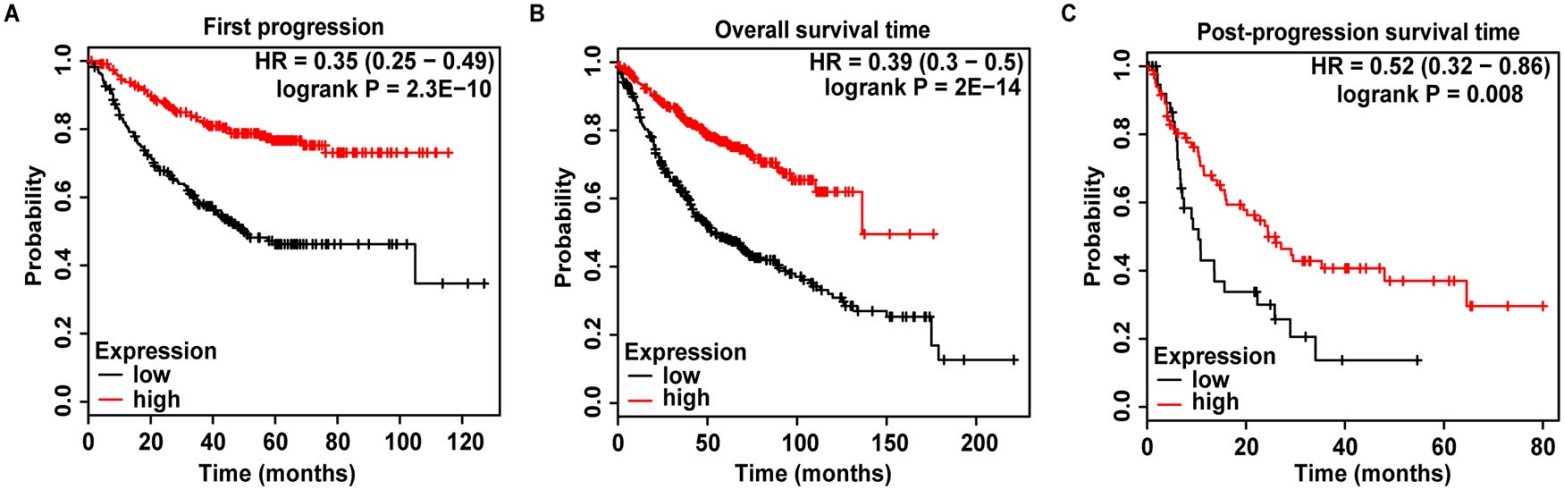
Analysis of ADARB1 expression on the prognosis of LUAD patients. (A-C) The relationship between ADARB1 expression and FS, OS or PPS, described by Kaplan-Meier Plotter, respectively.

After verifying the status of ADARB1 expression in LUAD, we further investigated the relationship between ADARB1 expression and the clinical characteristics of LUAD patients. The clinical data of LUAD patients was obtained from the Wanderer database, and the results showed that ADARB1 expression was significantly associated with gender (P = 0.001) and pathologic M stage (P = 0.000) (Table 1). Furthermore, the result of multivariate analysis revealed that sex (P = 0.004) and pathologic M stage (P = 0.000) obviously affected ADARB1 expression in LUAD patients (Table 2). Then, a Kaplan-Meier Plotter database was employed to evaluate the effects of ADARB1 expression on survival, which demonstrated that low levels of ADARB1 expression were correlated with shorter FP (P = 2.3E-10), OS (P = 2E-14) and PPS (P = 0.008) (Fig 4A–4C). Therefore, ADARB1 was supposed as a potential biomarker for clinical treatment and prognosis.

**Table 1:** Correlation between clinical characteristic parameters and ADARB1 expression in LUAD.

Variables	Number	Mean ± SD	P
**Gender**			**0.001**
Male	208	8.426 ± 0.901	
Female	249	8.693 ± 0.781	
**Pathologic T**			0.123
T1/T1a/T1b	140	8.724 ± 0.795	
T2/T2a/T2b	256	8.504 ± 0.872	
T3	41	8.514 ± 0.902	
T4	18	8.539 ± 0.672	
TX	2	8.025 ± 0.474	
**Pathologic N**			0.37
N0	290	8.617 ± 0.820	
N1	85	8.442 ± 0.806	
N2	70	8.501 ± 1.005	
N3	2	8.784 ± 0.276	
NX	9	8.831 ± 0.822	
**Pathologic M**			**0**
M0	313	8.489 ± 0.858	
M1/M1a/M1b	22	8.201 ± 0.872	
MX	118	8.860 ± 0.745	
**Pathologic tumor stage**			0.229
Stage I/IA/IB	246	8.606 ± 0.812	
Stage IIA/IIB	108	8.609 ± 0.851	
Stage IIIA/IIIB	79	8.509 ± 0.935	
Stage IV	23	8.251 ± 0.885	
**Race**			0.563
WHITE	356	8.612 ± 0.846	
BLACK OR AFRICAN AMERICAN	26	8.428 ± 0.887	
ASIAN	7	8.633 ± 0.791	
**Age**			0.171
≤60	137	8.491 ± 0.897	
>60	301	8.611 ± 0.830	
**Residual tumor**			0.209
RX	17	8.609 ± 0.681	
R0	315	8.489 ± 0.836	
R1	11	8.985 ± 0.698	
R2	4	8.233 ± 0.484	
**Tobacco smoking history indicator**			0.297
Current reformed smoker for > 15 years	115	8.590 ± 0.764	
Current reformed smoker for < or = 15 years	153	8.574 ± 0.845	
Current Reformed Smoker, Duration Not Specified	2	8.185 ± 1.722	
Lifelong Non-smoker	69	8.738 ± 0.806	
Current smoker	106	8.464 ± 0.916	

**Table 2:** Clinical multivariate data related to ADARB1 expression in LUAD.

Source	Type III Sum of Squares	df	Mean Square	F	p-value
Gender	5.733	1	5.733	8.437	0.004
Pathologic M	12.622	2	6.311	9.288	0.000

### Correlation between ADARB1 methylation and the clinical characteristics of LUAD patients

**Fig 5:**
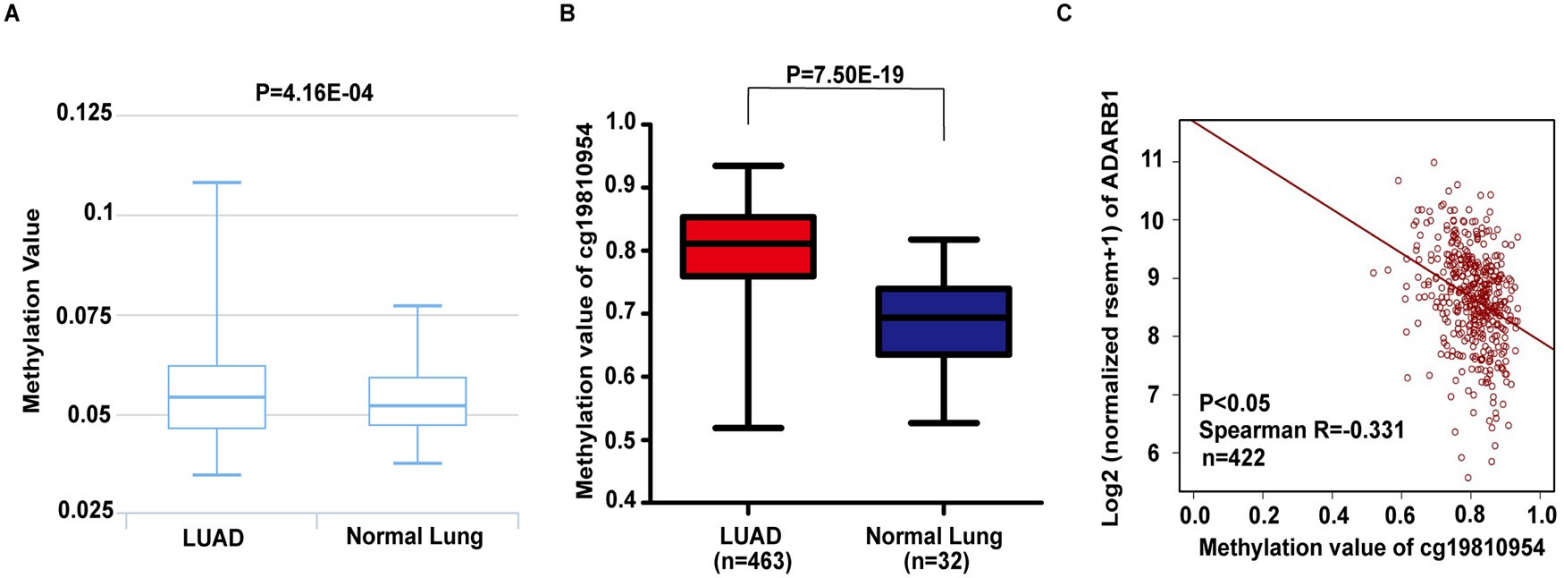
Methylation values of ADARB1 in LUAD patients. (A) Global ADARB1 methylation in LUAD samples compared with that in normal samples was analyzed by the DiseaseMeth version 2.0 database. (B) The data acquired from the Wanderer database indicated the highest methylation value of cg19810954 in ADARB1. (C) Negative correlations between cg19810954 methylation and the ADARB1 expression level.

Increasing evidence has demonstrated that DNA methylation plays a promising role in gene regulation in mammalian cells[33]. By using the DiseaseMeth version 2.0 database, we investigated the global methylation of ADARB1 in LUAD samples and found that ADARB1 was hypermethylated in LUAD samples compared with that in normal lung samples (P = 4.16E-04) (Fig 5A). Then, we screened out the highest methylation value of cg19810954 in ADARB1 from the data acquired from the Wanderer database (P = 7.50E-19) (Fig 5B, S2 Table). Meanwhile, Spearman’s linear correlation analysis indicated the negative correlation between the methylation value of cg19810954 and the ADARB1 expression level (P < 0.05) (Fig 5C), which further confirmed the low expression of ADARB1 in LUAD. The relationship between cg19810954 methylation and clinical characteristics of LUAD patients was conducted, and the results considered that the methylation value of cg19810954 was significantly related to the histologic diagnosis (P = 0.033) and ECOG score (P = 0.029), while the sample size of histologic diagnosis with lung bronchioloalveolar carcinoma mucinous and lung micropapillary adenocarcinoma were relative small, which may cause bias (n = 4; n = 3) (Table 3). Moreover, the histologic diagnosis (P = 0.000) and ECOG score (P = 0.017) were two independent factors that were influenced by the values of cg19810954 in LUAD patients in the multivariate analysis (Table 4).

**Table 3:** Correlation between clinical characteristics and the methylation value of cg19810954 in ADARB1 in LUAD patients.

Variables	Number	Mean ± SD	P
**Gender**			0.487
male	189	0.811 ± 0.072	
female	219	0.806 ± 0.067	
**Vital status**			0.863
Alive	311	0.808 ± 0.070	
Dead	97	0.809 ± 0.067	
**Kras mutation found**			0.866
YES	16	0.801 ± 0.074	
NO	34	0.797 ± 0.071	
**Pathologic T**			0.910
T1/T1a/T1b	127	0.810 ± 0.063	
T2/T2a/T2b	227	0.810 ± 0.069	
T3	36	0.794 ± 0.089	
T4	15	0.809 ± 0.072	
TX	3	0.788 ± 0.083	
**Pathologic N**			0.690
N0	261	0.806 ± 0.073	
N1	75	0.816 ± 0.057	
N2	62	0.810 ± 0.069	
N3	1	0.855	
NX	8	0.793 ± 0.048	
**Pathologic M**			0.552
M0	264	0.810 ± 0.068	
M1/M1a/M1b	17	0.815 ± 0.066	
MX	123	0.803 ± 0.071	
**Pathologic tumor stage**			0.799
Stage I/IA/IB	218	0.811 ± 0.069	
Stage IIA/IIB	102	0.803 ± 0.070	
Stage IIIA/IIIB	68	0.809 ± 0.069	
Stage IV	19	0.807 ± 0.068	
**Race**			0.996
WHITE	325	0.809 ± 0.069	
BLACK OR AFRICAN AMERICAN	29	0.808 ± 0.066	
ASIAN	5	0.810 ± 0.041	
**Histologic diagnosis**			**0.033**
Lung Adenocarcinoma Mixed Subtype	79	0.830 ± 0.058	
Lung Adenocarcinoma- Not Otherwise Specified (NOS)	259	0.805 ± 0.068	
Lung Mucinous Adenocarcinoma	2	0.766 ± 0.100	
Lung Acinar Adenocarcinoma	13	0.802 ± 0.070	
Lung Bronchioloalveolar Carcinoma Mucinous	4	0.850 ± 0.010	
Lung Papillary Adenocarcinoma	17	0.819 ± 0.059	
Lung Micropapillary Adenocarcinoma	3	0.745 ± 0.114	
Mucinous (Colloid) Carcinoma	7	0.776 ± 0.090	
Lung Bronchioloalveolar Carcinoma Nonmucinous	19	0.784 ± 0.098	
Lung Solid Pattern Predominant Adenocarcinoma	4	0.794 ± 0.082	
**Age**			0.845
≤60	131	0.809 ± 0.072	
>60	259	0.808 ± 0.067	
**ECOG score**			**0.029**
0	79	0.804 ± 0.067	
1	68	0.832 ± 0.061	
2	21	0.811 ± 0.064	

**Table 4:** Clinical multivariate data related to the methylation value of cg19810954 in ADARB1 in LUAD.

Source	Type III Sum of Squares	df	Mean Square	F	p-value
Histologic diagnosis	0.130	9	0.014	4.129	0.000
ECOG score	0.029	2	0.015	4.167	0.017

### Correlation between ADARB1 methylation and LUAD cell metastasis

**Fig 6:**
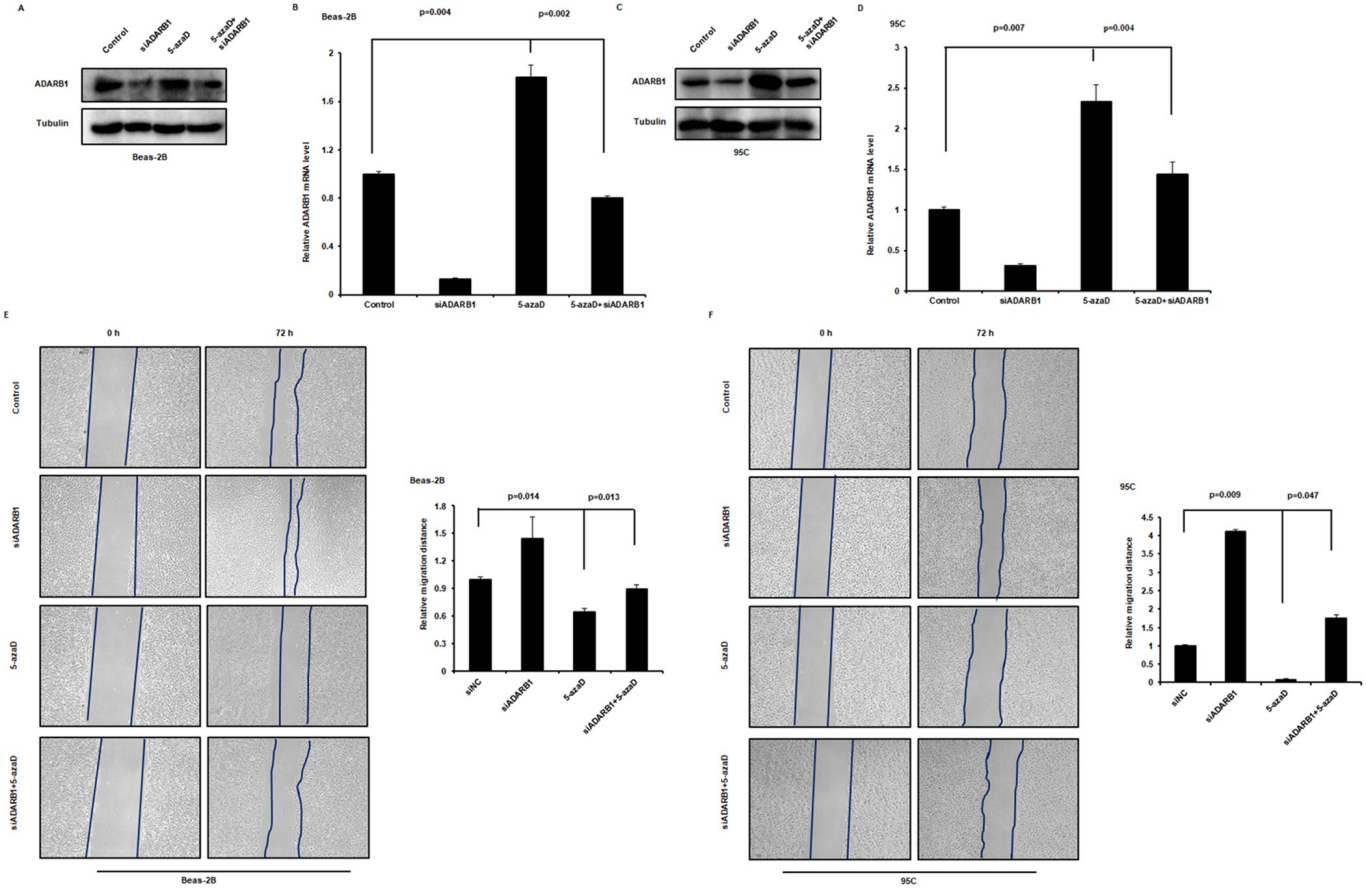
Modulation of DNA methylation inhibited ADARB1-reduced LUAD cell metastasis. Beas-2B (A, B) and 95C cells (C, D) treated with siADARB1 and/or DNMT inhibitors 5-azaD subjected to Western blot and RT-PCR assays. The wound healing assays were performed after treating Beas-2B (E) and 95C (F) cells with siADARB1 and/or DNMT inhibitors 5-azaD.

DNA methylation is a biological process accomplished by DNA methyltransferases (DNMTs), which catalyze a covalent addition of a methyl group to the 5-position of cytosine within the CpGs island[34]. To investigate whether ADARB1 methylation levels is associated with metastasis of LUAD cells, we examined the expression of ADARB1 after DNMT inhibitors 5-aza-2-deoxycytidine (5-azaD) treatment in two lung cancer cell lines. We observed that the expression of ADARB1 was higher in 5-azaD+siADARB1 compared to siADARB1 cells and showed that inactivation of DNA methylation using inhibitors 5-azaD could elevate the siRNA-mediated downregulation of ADARB1 in Beas-2B and 95C cell lines (Fig 6A–6D). To evaluate whether the effect of ADARB1 was through DNA methylation mechanism, we examined cell migration capacity of Beas-2B and 95C cells under siADARB1 transfection and 5-azaD treatment. Compared to the non-treatment group, lung cancer cells showed a significantly lower migration distance when treated with inhibitors 5-azaD. Similar results were obtained after combinational treatment, and the promotion effects of siADARB1 were remarkably decreased by 5-azaD treatment in Beas-2B and 95C cell lines (Fig 6E and 6F). These finding further supports the notion that promoting-cancer activity by ADARB1 inhibition is a consequence of the inhibition of DNA methylation signaling.

### Functional enrichment analysis of ADARB1-associated coexpression genes

**Fig 7:**
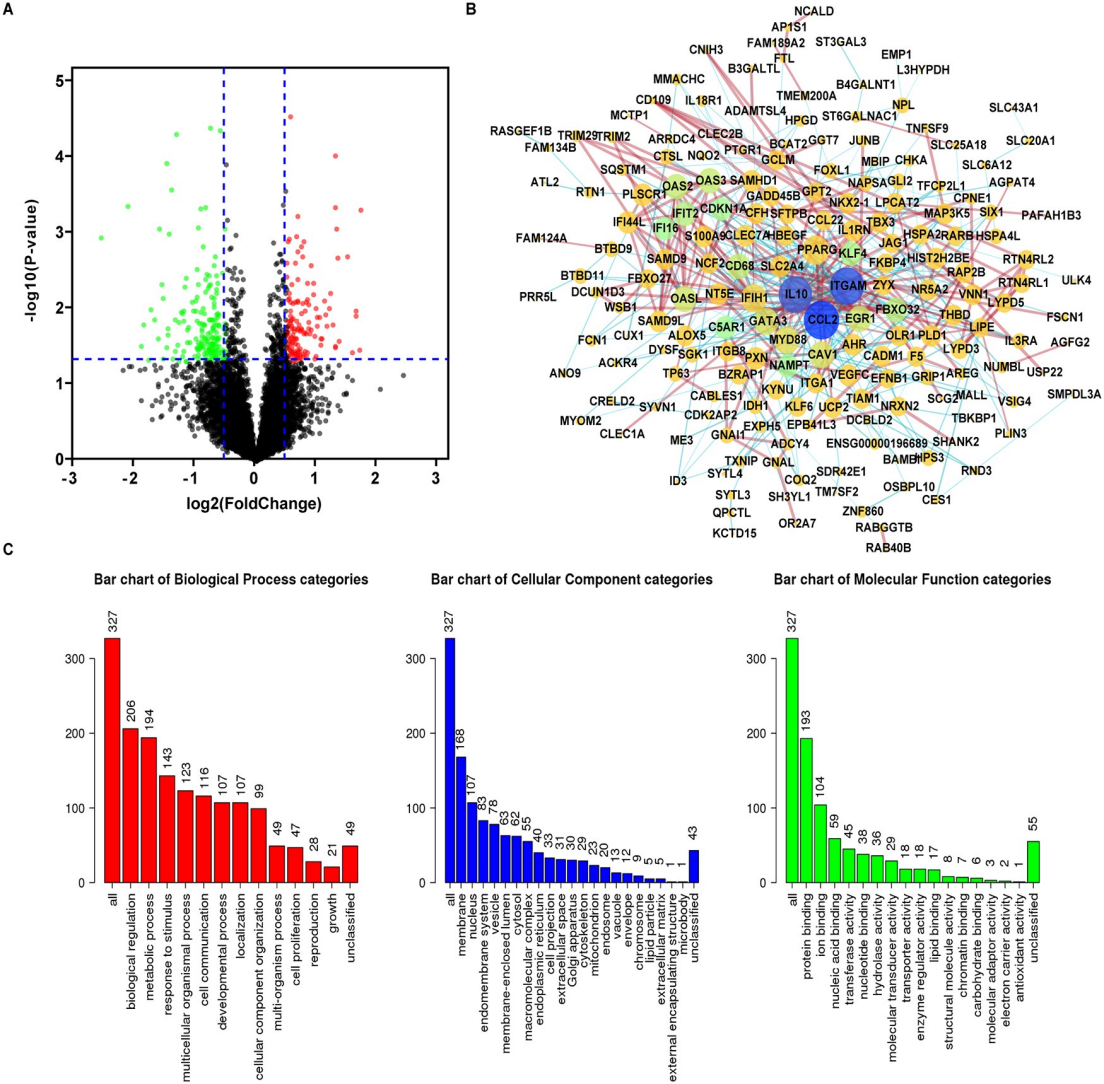
Functional enrichment analysis of ADARB1-associated co-DEGs in LUAD samples. (A) The coexpression genes of ADARB1 are shown as a volcano plot. (B) The PPI network of ADARB1-associated co-DEGs as completed by the STRING and Cytoscape software. (C) GO analysis of ADARB1 associated co-DEGs.

Finally, PPI analysis of ADARB1-associated coexpressed genes was performed for the purpose of understanding the biological function of ADARB1. Through the cBioPortal database, 13740 genes significantly coexpressing with ADARB1 in LUAD samples were obtained. Then, we constructed a volcano plot to show the grouping between altered and unaltered ADARB1 expression (Fig 7A). According to the criteria of p value < 0.05 and |log Ratio| > 0.5, 332 genes were selected as ADARB1-associated codifferentially expressed genes (co-DEGs) (S3 Table). Subsequently, a PPI network was built by using the STRING database and Cytoscape software (Fig 7B). At the same time, GO (Fig 7C) and KEGG analysis were conducted using WebGestalt and DAVID web tools, respectively. As shown in the figure, the biological processes indicated that the co-DEGs were mainly concentrated on biological regulation and metabolic processes. For cellular components, the cell membrane and nucleus were more preferred by the co-DEGs. In the aspect of molecular function, the co-DEGs were primarily enriched in protein binding and ion binding. Moreover, the KEGG pathway demonstrated that these genes were significantly related to oxocarboxylic acid metabolism, as shown in S4 Table.

## Discussion

Our study was the first to study the expression and function of ADARB1 in LUAD and its association with clinical characteristics from a bioinformatics viewpoint. The results confirmed that ADARB1 was markedly decreased in LUAD tissues and cell lines. Patients with low ADARB1 expression always had shorter FP, OS and PPS.

ADARB1 is an RNA editase that catalyzes A-I deamination in double-stranded regions, is ubiquitously expressed in many tissues, and is involved in many diseases[35]. Recent studies have indicated the possible role of ADARB1 in the development of cancer. Most reports have linked cancer with reduced ADARB1 expression or activity. By editing and stabilizing insulin-like growth factor binding protein 7 (IGFBP7), ADARB1 overexpression suppressed tumor growth and induced apoptosis in esophageal squamous cell carcinoma[36]. The ADARB1 alternative splicing variant (ASV) might be correlated with the invasiveness of gliomas[37]. However, a clustering analysis based on gene function showed that ADARB1 was upregulated in prostate cancer (PCa) tissues[38]. These inconsistent conclusions might be due to different disease status with diverse pathological states, suggesting randomized controlled, international, multicenter clinical research is needed for further study.

In the present study, ADARB1 expression was significantly lower in metastatic LUAD cells. The results showed that ADARB1 might play an essential role in inhibiting metastasis. In accordance with our findings, previous studies have demonstrated that the PKCζ/ADARB1 axis is a critical regulator of colorectal cancer metastases through regulating RNA editing mediated miR-200 secretion[39]. Moreover, the ADARB1/miR-589-3p axis has been proven to inhibit glioblastoma cell migration and invasion[40]. However, no statically difference was found between ADARB1 expression and lymph nodes metastasis, though experiments displayed significantly increased migration distance with siADARB1 treatment, which needed further study. Additionally, the analysis of ADARB1-associated co-DEGs suggested several potential regulators for ADARB1-regulated metastasis of lung cancer in our study. The molecules myeloid differentiation factor 88 (MyD88) is an adaptor protein of Toll-like receptor (TLR) signaling pathways that activates the innate immune system[41]. Studies have found that MRSA infection can enhance NSCLC cell metastasis by up-regulating TLR4/MyD88 signaling[42]. JAG1 is a Notch ligand that makes a difference in various signaling pathways. Chang et al. discovered that JAG1 was a potential metastasis enhancer in lung cancer and JAG1/HSPA2 axis mediated lung cancer malignancy[43]. CCL2 is one of cytokine genes that involves in immunoregulatory and inflammatory processes. Endothelial cells activation was facilitated by CCL2-CCR2 signaling through myosin light chain phosphorylation, which enhanced tumor cell migration and metastasis[44]. According to our study, by targeting these positive and negative metastasis-associated genes, future experiments will verify these above hypotheses with a larger sample size, and standardize the sampling and processing process to reduce bias.

## Conclusion

In summary, our study found that ADARB1 could be used as a promising biomarker both in tumorigenesis and treatment of LUAD patients. A better understanding of its potential roles and mechanisms in LUAD biology is of great significance for the study of the prognosis and therapy.

## Supporting information

S1 Table Main bioinformatics tools applied to analyze the roles of ADARB1 in LUAD biological processes. (DOCX)

S2 Table Methylation values of CpG islands in ADARB1. (DOCX)

S3 Table ADARB1-associated co-DEGs in LUAD patients. (DOCX)

S4 Table KEGG pathway of ADARB1-associated co-DEGs in LUAD patients. (DOCX)
