# Supplementary material for: Function of low ADARB1 expression in lung adenocarcinoma
Source: PLoS One. 2019 Sep 6;14(9):e0222298. doi: 10.1371/journal.pone.0222298 (PMC6730894; doi:10.1371/journal.pone.0222298)
Supplement: S1 Table — (DOCX) [file pone.0222298.s001.docx]

Supplemental table 1 Main bioinformatics tools applied to analyze the roles of ADARB1 in LUAD biological processes.

| **Databases** | **URL** | **Refs** |
| --- | --- | --- |
| Oncomine | https://www.oncomine.org/resource/login.html | [10] |
| CCLE | <https://portals.broadinstitute.org/ccle/about> | [16] |
| UALCAN | <http://ualcan.path.uab.edu/index.html> | [17] |
| GEPIA | http://gepia.cancer-pku.cn/ | [18] |
| GE-mini | http://gemini.cancer-pku.cn/ | [19] |
| CRN | <http://syslab4.nchu.edu.tw/> | [20] |
| GEO | https://www.ncbi.nlm.nih.gov/geoprofiles/ | [21] |
| Wanderer | http://maplab.imppc.org/wanderer/ | [22] |
| Kaplan-Meier plotter | http://kmplot.com/analysis/ | [23] |
| DiseaseMeth | <https://cancergenome.nih.gov/> | [24] |
| cBioPortal | http://www.cbioportal.org/ | [25] |
| STRING | https://string-db.org/cgi/input.pl | [27] |
| Cytoscape | https://cytoscape.org/ | [28] |
| KEGG | https://www.kegg.jp/ | [29] |
| WebGestalt | http://www.webgestalt.org/ | [30] |
| DAVID | https://david.ncifcrf.gov/ | [31] |
