# Supplementary material for: Function of low ADARB1 expression in lung adenocarcinoma
Source: PLoS One. 2019 Sep 6;14(9):e0222298. doi: 10.1371/journal.pone.0222298 (PMC6730894; doi:10.1371/journal.pone.0222298)
Supplement: S2 Table — (DOCX) [file pone.0222298.s002.docx]

Supplemental table 2 Methylation values of CpG islands in ADARB1.

| **probe** | **chr** | **cg-start** | **cg-end** | **percentgc** | **Tum-mean** | **Tum sd** | **Wilcox-stat** | **P-value** |
| --- | --- | --- | --- | --- | --- | --- | --- | --- |
| cg19810954 | chr21 | 46496510 | 46496511 | 0.46 | 0.803122 | 0.070225 | 1598 | 1.14E-13 |
| cg05516004 | chr21 | 46496180 | 46496181 | 0.42 | 0.873944 | 0.033913 | 1909 | 2.12E-12 |
| cg05575217 | chr21 | 46518030 | 46518031 | 0.62 | 0.835183 | 0.105353 | 2727 | 2.22E-09 |
| cg23662138 | chr21 | 46495607 | 46495608 | 0.44 | 0.290482 | 0.172887 | 2952 | 1.24E-08 |
| cg04233620 | chr21 | 46629219 | 46629220 | 0.4 | 0.750571 | 0.130042 | 11513 | 1.56E-07 |
| cg00927699 | chr21 | 46493289 | 46493290 | 0.56 | 0.739432 | 0.120372 | 3581 | 1.06E-06 |
| cg22635096 | chr21 | 46550644 | 46550645 | 0.52 | 0.462321 | 0.168969 | 11034 | 3.61E-06 |
| cg11987819 | chr21 | 46495389 | 46495390 | 0.7 | 0.042551 | 0.076237 | 3911 | 7.89E-06 |
| cg14526825 | chr21 | 46646076 | 46646077 | 0.48 | 0.534016 | 0.153446 | 10751 | 1.94E-05 |
| cg16358576 | chr21 | 46644306 | 46644307 | 0.6 | 0.882912 | 0.095962 | 10322 | 0.000197 |
| cg01603980 | chr21 | 46521050 | 46521051 | 0.66 | 0.800111 | 0.06931 | 10236 | 0.000302 |
| cg09676390 | chr21 | 46493345 | 46493346 | 0.56 | 0.467014 | 0.110489 | 9832 | 0.001956 |
| cg25675393 | chr21 | 46494274 | 46494275 | 0.8 | 0.0214 | 0.004594 | 5154 | 0.003981 |
| cg14194835 | chr21 | 46640872 | 46640873 | 0.62 | 0.972084 | 0.052951 | 9598.5 | 0.005134 |
| cg06000635 | chr21 | 46492095 | 46492096 | 0.52 | 0.761653 | 0.097813 | 9430 | 0.009789 |
| cg27449258 | chr21 | 46493436 | 46493437 | 0.48 | 0.598926 | 0.178606 | 9089 | 0.031758 |
| cg18570853 | chr21 | 46557241 | 46557242 | 0.62 | 0.344536 | 0.113889 | 9029 | 0.03838 |
| cg15885953 | chr21 | 46520883 | 46520884 | 0.7 | 0.964545 | 0.070538 | 8962 | 0.047127 |
| cg12703219 | chr21 | 46556392 | 46556393 | 0.56 | 0.764316 | 0.101049 | 8811 | 0.0731 |
| cg18501409 | chr21 | 46553607 | 46553608 | 0.52 | 0.923991 | 0.033278 | 6063.5 | 0.085897 |
| cg23919038 | chr21 | 46554352 | 46554353 | 0.68 | 0.919808 | 0.017476 | 8668 | 0.107514 |
| cg25894939 | chr21 | 46521281 | 46521282 | 0.66 | 0.917159 | 0.099188 | 8354 | 0.226963 |
| cg08222185 | chr21 | 46494823 | 46494824 | 0.72 | 0.028398 | 0.010902 | 7085 | 0.680258 |
| cg24063645 | chr21 | 46553953 | 46553954 | 0.72 | 0.986146 | 0.010209 | 7646.5 | 0.761028 |
| cg11424525 | chr21 | 46494378 | 46494379 | 0.68 | 0.070655 | 0.014722 | 7490 | 0.917054 |
| cg21217129 | chr21 | 46554127 | 46554128 | 0.66 | 0.848033 | 0.041269 | 7416 | 0.992353 |
