# Supplementary material for: Function of low ADARB1 expression in lung adenocarcinoma
Source: PLoS One. 2019 Sep 6;14(9):e0222298. doi: 10.1371/journal.pone.0222298 (PMC6730894; doi:10.1371/journal.pone.0222298)
Supplement: S3 Table — (DOCX) [file pone.0222298.s003.docx]

Supplemental table 3 ADARB1-associated co-DEGs in LUAD patients.

| **Gene** | **Cytoband** | **Log Ratio** | **p-Value** | **q-Value** | **Tendency** |
| --- | --- | --- | --- | --- | --- |
| ZBTB3 | 11q12.3 | 0.56 | 2.82E-05 | 0.168 | Over-expressed |
| CCDC71L | 7q22.3 | -0.76 | 4.03E-05 | 0.168 | Under-expressed |
| MYD88 | 3p22.2 | -0.6 | 4.38E-05 | 0.168 | Under-expressed |
| GRAMD1B | 11q24.1 | -1.32 | 4.90E-05 | 0.168 | Under-expressed |
| NKX2-1 | 14q13.3 | 1.31 | 9.28E-05 | 0.246 | Over-expressed |
| ATP8B3 | 19p13.3 | -1.48 | 1.20E-04 | 0.246 | Under-expressed |
| PXN | 12q24.23 | -0.5 | 1.25E-04 | 0.246 | Under-expressed |
| LYPD3 | 19q13.31 | -1.39 | 2.62E-04 | 0.416 | Under-expressed |
| TNS4 | 17q21.2 | -2.12 | 4.42E-04 | 0.447 | Under-expressed |
| LARGE2 | 11p11.2 | 1.31 | 4.52E-04 | 0.447 | Over-expressed |
| ARRDC4 | 15q26.2 | -0.84 | 4.59E-04 | 0.447 | Under-expressed |
| FAM124A | 13q14.3 | -0.92 | 4.82E-04 | 0.447 | Under-expressed |
| RIMKLA | 1p34.2 | 1.72 | 4.88E-04 | 0.447 | Over-expressed |
| HOOK1 | 1p32.1 | 0.69 | 5.87E-04 | 0.504 | Over-expressed |
| CRYBG1 | 6q21 | -0.95 | 8.45E-04 | 0.582 | Under-expressed |
| PLIN3 | 19p13.3 | -0.52 | 8.60E-04 | 0.582 | Under-expressed |
| ST6GALNAC1 | 17q25.1 | 1.33 | 8.73E-04 | 0.582 | Over-expressed |
| TRIM7 | 5q35.3 | -1.59 | 8.90E-04 | 0.582 | Under-expressed |
| NPTXR | 22q13.1 | -1.44 | 1.00E-03 | 0.622 | Under-expressed |
| SS18L1 | 20q13.33 | 0.66 | 1.10E-03 | 0.622 | Over-expressed |
| TRIM29 | 11q23.3 | -2.55 | 1.16E-03 | 0.622 | Under-expressed |
| CRELD2 | 22q13.33 | 0.54 | 1.18E-03 | 0.622 | Over-expressed |
| MMACHC | 1p34.1 | 0.53 | 1.28E-03 | 0.631 | Over-expressed |
| TSPOAP1 | 17q22 | 0.79 | 1.29E-03 | 0.631 | Over-expressed |
| PTK7 | 6p21.1 | 0.75 | 1.48E-03 | 0.661 | Over-expressed |
| CTSL | 9q21.33 | -0.87 | 1.49E-03 | 0.661 | Under-expressed |
| ZNF569 | 19q13.12 | 0.69 | 1.72E-03 | 0.694 | Over-expressed |
| SAMD9 | 7q21.2 | -0.68 | 2.03E-03 | 0.725 | Under-expressed |
| TBC1D8 | 2q11.2 | 0.77 | 2.03E-03 | 0.725 | Over-expressed |
| RTN4RL2 | 11q12.1 | 1.5 | 2.06E-03 | 0.725 | Over-expressed |
| CADM1 | 11q23.3 | 1.35 | 2.14E-03 | 0.736 | Over-expressed |
| EXPH5 | 11q22.3 | 0.51 | 2.43E-03 | 0.75 | Over-expressed |
| ZNF248 | 10p11.21 | 0.64 | 2.46E-03 | 0.75 | Over-expressed |
| IFIT2 | 10q23.31 | -0.7 | 2.67E-03 | 0.775 | Under-expressed |
| SMPDL3A | 6q22.31 | 0.9 | 2.71E-03 | 0.775 | Over-expressed |
| NR5A2 | 1q32.1 | -0.97 | 3.00E-03 | 0.808 | Under-expressed |
| FKBP4 | 12p13.33 | -0.56 | 3.35E-03 | 0.83 | Under-expressed |
| FSCN1 | 7p22.1 | -0.82 | 3.51E-03 | 0.836 | Under-expressed |
| CARD6 | 5p13.1 | -0.78 | 3.62E-03 | 0.836 | Under-expressed |
| IDH1 | 2q34 | -0.65 | 3.63E-03 | 0.836 | Under-expressed |
| ZNF821 | 16q22.2 | 0.51 | 3.75E-03 | 0.836 | Over-expressed |
| VNN1 | 6q23.2 | -1.15 | 3.96E-03 | 0.836 | Under-expressed |
| SLC25A18 | 22q11.21 | -0.83 | 4.00E-03 | 0.836 | Under-expressed |
| SSR4 | Xq28 | 0.68 | 4.07E-03 | 0.836 | Over-expressed |
| NPL | 1q25.3 | -0.64 | 4.48E-03 | 0.86 | Under-expressed |
| ETV4 | 17q21.31 | 0.95 | 4.49E-03 | 0.86 | Over-expressed |
| DHRS13 | 17q11.2 | 0.84 | 4.61E-03 | 0.86 | Over-expressed |
| OASL | 12q24.31 | -0.95 | 4.66E-03 | 0.86 | Under-expressed |
| RAB40B | 17q25.3 | 0.59 | 4.70E-03 | 0.86 | Over-expressed |
| CDCP1 | 3p21.31 | -0.75 | 4.71E-03 | 0.86 | Under-expressed |
| RETREG1 | 5p15.1 | -1.56 | 4.78E-03 | 0.86 | Under-expressed |
| IFI16 | 1q23.1 | -0.77 | 4.83E-03 | 0.86 | Under-expressed |
| CAMK2N1 | 1p36.12 | -1.18 | 5.12E-03 | 0.862 | Under-expressed |
| EFNB1 | Xq13.1 | -0.79 | 5.37E-03 | 0.862 | Under-expressed |
| RABGGTB | 1p31.1 | 0.5 | 5.51E-03 | 0.862 | Over-expressed |
| CLEC7A | 12p13.2 | -0.66 | 5.52E-03 | 0.862 | Under-expressed |
| JAG1 | 20p12.2 | -0.84 | 5.97E-03 | 0.898 | Under-expressed |
| ZNF233 | 19q13.31 | 1.23 | 6.17E-03 | 0.898 | Over-expressed |
| CYB5RL | 1p32.3 | 0.55 | 6.22E-03 | 0.898 | Over-expressed |
| HTATIP2 | 11p15.1 | -0.66 | 6.24E-03 | 0.898 | Under-expressed |
| GGT7 | 20q11.22 | 0.95 | 6.50E-03 | 0.901 | Over-expressed |
| CNIH3 | 1q42.12 | -0.74 | 6.89E-03 | 0.901 | Under-expressed |
| HSPA2 | 14q23.3 | -0.97 | 6.98E-03 | 0.901 | Under-expressed |
| ZFP28 | 19q13.43 | 0.54 | 7.16E-03 | 0.901 | Over-expressed |
| TINCR | 19p13.3 | -1.53 | 7.20E-03 | 0.901 | Under-expressed |
| AHR | 7p21.1 | -0.7 | 7.28E-03 | 0.901 | Under-expressed |
| ME3 | 11q14.2 | 0.84 | 7.50E-03 | 0.901 | Over-expressed |
| CDRT4 | 17p12 | 0.52 | 7.63E-03 | 0.901 | Over-expressed |
| FAM189A2 | 9q21.12 | 1.13 | 7.66E-03 | 0.901 | Over-expressed |
| ZNF879 | 5q35.3 | 0.69 | 7.69E-03 | 0.901 | Over-expressed |
| ULK4 | 3p22.1 | -1 | 7.77E-03 | 0.901 | Under-expressed |
| WSB1 | 17q11.1 | 0.83 | 8.09E-03 | 0.901 | Over-expressed |
| PWARSN | 15q11.2 | 0.57 | 8.13E-03 | 0.901 | Over-expressed |
| AGPAT4 | 6q26 | -1.05 | 8.18E-03 | 0.901 | Under-expressed |
| ZNF607 | 19q13.12 | 0.64 | 8.20E-03 | 0.901 | Over-expressed |
| ZNF568 | 19q13.12 | 0.69 | 8.24E-03 | 0.901 | Over-expressed |
| NINL | 20p11.21 | 0.77 | 8.44E-03 | 0.901 | Over-expressed |
| TOX2 | 20q13.12 | -0.69 | 8.46E-03 | 0.901 | Under-expressed |
| MXD4 | 4p16.3 | 0.6 | 8.52E-03 | 0.901 | Over-expressed |
| CMAHP | 6p22.3 | 0.86 | 8.87E-03 | 0.909 | Over-expressed |
| SOWAHC | 2q13 | -0.69 | 9.55E-03 | 0.958 | Under-expressed |
| HIST2H2BE | 1q21.2 | -0.88 | 9.63E-03 | 0.958 | Under-expressed |
| SLC43A1 | 11q12.1 | 1.21 | 9.72E-03 | 0.961 | Over-expressed |
| MALL | 2q13 | 0.98 | 9.86E-03 | 0.967 | Over-expressed |
| TP63 | 3q28 | -1.74 | 0.01 | 0.975 | Under-expressed |
| ZSCAN9 | 6p22.1 | 0.57 | 0.0104 | 0.99 | Over-expressed |
| SP6 | 17q21.32 | -1.04 | 0.0105 | 0.99 | Under-expressed |
| MTURN | 7p14.3 | 0.81 | 0.0106 | 0.99 | Over-expressed |
| VEGFC | 4q34.3 | -0.99 | 0.0107 | 0.99 | Under-expressed |
| KYNU | 2q22.2 | -1.2 | 0.0107 | 0.99 | Under-expressed |
| FAM149A | 4q35.1 | 1.64 | 0.0107 | 0.991 | Over-expressed |
| NAMPT | 7q22.3 | -0.92 | 0.011 | 1 | Under-expressed |
| LRRC6 | 8q24.22 | -1.24 | 0.0111 | 1 | Under-expressed |
| LOC220729 | 3q29 | -0.63 | 0.0113 | 1 | Under-expressed |
| TNFRSF6B | 20q13.33 | -1.22 | 0.0113 | 1 | Under-expressed |
| DNAJC28 | 21q22.11 | 0.75 | 0.0115 | 1 | Over-expressed |
| TIAM1 | 21q22.11 | -0.86 | 0.0115 | 1 | Under-expressed |
| CLEC1A | 12p13.2 | -0.61 | 0.0117 | 1 | Under-expressed |
| CD68 | 17p13.1 | -0.66 | 0.0117 | 1 | Under-expressed |
| TRIM2 | 4q31.3 | 1.14 | 0.012 | 1 | Over-expressed |
| NAPSA | 19q13.33 | 1.64 | 0.0122 | 1 | Over-expressed |
| SPCS2 | 11q13.4 | 0.57 | 0.0125 | 1 | Over-expressed |
| CDK2AP2 | 11q13.2 | 1.04 | 0.0126 | 1 | Over-expressed |
| SIX1 | 14q23.1 | 1.01 | 0.0127 | 1 | Over-expressed |
| EGR1 | 5q31.2 | -0.63 | 0.0128 | 1 | Under-expressed |
| MAP4K2 | 11q13.1 | 0.6 | 0.0133 | 1 | Over-expressed |
| OR2A7 | 7q35 | -0.74 | 0.0137 | 1 | Under-expressed |
| GADD45B | 19p13.3 | -0.71 | 0.0137 | 1 | Under-expressed |
| RTN1 | 14q23.1 | -0.75 | 0.0138 | 1 | Under-expressed |
| TRIM16 | 17p12 | -0.77 | 0.0138 | 1 | Under-expressed |
| HSPA4L | 4q28.1 | -1.68 | 0.0138 | 1 | Under-expressed |
| HPGD | 4q34.1 | -1.34 | 0.0139 | 1 | Under-expressed |
| UST | 6q25.1 | -0.68 | 0.0139 | 1 | Under-expressed |
| EDRF1 | 10q26.2 | 0.59 | 0.0141 | 1 | Over-expressed |
| MYOM2 | 8p23.3 | -0.97 | 0.0142 | 1 | Under-expressed |
| ZNF774 | 15q26.1 | 0.57 | 0.0142 | 1 | Over-expressed |
| MTMR9LP | 1p35.2 | 0.66 | 0.0144 | 1 | Over-expressed |
| SPCS2P4 | 1p35.3 | 0.52 | 0.0144 | 1 | Over-expressed |
| S100A9 | 1q21.3 | -1.2 | 0.0146 | 1 | Under-expressed |
| PLEKHH2 | 2p21 | 0.87 | 0.0149 | 1 | Over-expressed |
| ASPH | 8q12.3 | -1.28 | 0.0149 | 1 | Under-expressed |
| PIR | Xp22.2 | -0.89 | 0.0154 | 1 | Under-expressed |
| TMEM79 | 1q22 | -0.51 | 0.0157 | 1 | Under-expressed |
| ZDHHC9 | Xq26.1 | 0.56 | 0.0157 | 1 | Over-expressed |
| TPRG1 | 3q28 | -0.94 | 0.0158 | 1 | Under-expressed |
| TMEM71 | 8q24.22 | -0.7 | 0.0159 | 1 | Under-expressed |
| CFH | 1q31.3 | -0.86 | 0.016 | 1 | Under-expressed |
| ANO9 | 11p15.5 | 0.7 | 0.0161 | 1 | Over-expressed |
| OAS3 | 12q24.13 | -0.86 | 0.0161 | 1 | Under-expressed |
| RAP2B | 3q25.2 | -0.62 | 0.0162 | 1 | Under-expressed |
| MBIP | 14q13.3 | 1.27 | 0.0163 | 1 | Over-expressed |
| F5 | 1q24.2 | -1.85 | 0.0163 | 1 | Under-expressed |
| AGFG2 | 7q22.1 | -0.66 | 0.0164 | 1 | Under-expressed |
| GCLM | 1p22.1 | -0.62 | 0.0165 | 1 | Under-expressed |
| ZDHHC16 | 10q24.1 | 0.53 | 0.0165 | 1 | Over-expressed |
| SDR42E1 | 16q23.3 | 1 | 0.0166 | 1 | Over-expressed |
| ZNF671 | 19q13.43 | 0.58 | 0.0167 | 1 | Over-expressed |
| B3GLCT | 13q12.3 | 0.53 | 0.017 | 1 | Over-expressed |
| 1-Mar | 1q41 | 1.17 | 0.017 | 1 | Over-expressed |
| AGAP4 | 10q11.22 | 0.5 | 0.0172 | 1 | Over-expressed |
| PTGR1 | 9q31.3 | -0.71 | 0.0173 | 1 | Under-expressed |
| CD109 | 6q13 | -1.12 | 0.0177 | 1 | Under-expressed |
| TMEM50B | 21q22.11 | 0.68 | 0.0178 | 1 | Over-expressed |
| PRR5L | 11p13-p12 | -0.72 | 0.0178 | 1 | Under-expressed |
| ATAT1 | 6p21.33 | 0.63 | 0.0178 | 1 | Over-expressed |
| KLF4 | 9q31.2 | -1.46 | 0.018 | 1 | Under-expressed |
| NBR2 | 17q21.31 | 0.59 | 0.018 | 1 | Over-expressed |
| ZYX | 7q34 | -0.53 | 0.018 | 1 | Under-expressed |
| HPN | 19q13.11 | 0.87 | 0.018 | 1 | Over-expressed |
| TAPT1 | 4p15.32 | 0.75 | 0.0182 | 1 | Over-expressed |
| BTBD11 | 12q23.3 | -0.89 | 0.0184 | 1 | Under-expressed |
| THBD | 20p11.21 | -0.69 | 0.0187 | 1 | Under-expressed |
| OLR1 | 12p13.2 | -1.14 | 0.019 | 1 | Under-expressed |
| KCNIP4 | 4p15.31-p15.2 | 0.9 | 0.019 | 1 | Over-expressed |
| TM7SF2 | 11q13.1 | 0.75 | 0.019 | 1 | Over-expressed |
| RNLS | 10q23.31 | 0.69 | 0.0192 | 1 | Over-expressed |
| MFSD10 | 4p16.3 | 0.73 | 0.0193 | 1 | Over-expressed |
| LRRC40 | 1p31.1 | 0.51 | 0.0193 | 1 | Over-expressed |
| SLC6A12 | 12p13.33 | -0.9 | 0.0194 | 1 | Under-expressed |
| ATL2 | 2p22.2-p22.1 | 0.63 | 0.0195 | 1 | Over-expressed |
| LYPD5 | 19q13.31 | -0.75 | 0.02 | 1 | Under-expressed |
| ALG6 | 1p31.3 | 0.52 | 0.0204 | 1 | Over-expressed |
| PLSCR1 | 3q24 | -0.65 | 0.0205 | 1 | Under-expressed |
| GCAT | 22q13.1 | 0.82 | 0.0206 | 1 | Over-expressed |
| AGAP5 | 10q22.2 | 0.5 | 0.0207 | 1 | Over-expressed |
| SGK1 | 6q23.2 | -0.52 | 0.0211 | 1 | Under-expressed |
| CLEC2B | 12p13.31 | -0.5 | 0.0213 | 1 | Under-expressed |
| ITGAM | 16p11.2 | -0.8 | 0.0214 | 1 | Under-expressed |
| UCP2 | 11q13.4 | 0.78 | 0.0214 | 1 | Over-expressed |
| DANCR | 4q12 | 0.89 | 0.0215 | 1 | Over-expressed |
| SYVN1 | 11q13.1 | 0.51 | 0.0215 | 1 | Over-expressed |
| KCTD14 | 11q14.1 | 0.83 | 0.0215 | 1 | Over-expressed |
| TMEM246 | 9q31.1 | -1.46 | 0.0218 | 1 | Under-expressed |
| TRPV1 | 17p13.2 | 0.61 | 0.0219 | 1 | Over-expressed |
| STK31 | 7p15.3 | 0.97 | 0.0226 | 1 | Over-expressed |
| LGALS9C | 17p11.2 | -0.89 | 0.0229 | 1 | Under-expressed |
| BMS1P1 | 10q11.22 | 0.53 | 0.0231 | 1 | Over-expressed |
| SLC20A1 | 2q14.1 | -0.62 | 0.0234 | 1 | Under-expressed |
| RASAL2-AS1 | 1q25.2 | -0.68 | 0.0236 | 1 | Under-expressed |
| RTN4RL1 | 17p13.3 | -0.85 | 0.0237 | 1 | Under-expressed |
| NUMBL | 19q13.2 | -0.74 | 0.0238 | 1 | Under-expressed |
| ZNF503-AS2 | 10q22.2 | 0.58 | 0.0238 | 1 | Over-expressed |
| TCAF1 | 7q35 | -0.72 | 0.0241 | 1 | Under-expressed |
| B4GALNT1 | 12q13.3 | -1.6 | 0.0243 | 1 | Under-expressed |
| PABPC1L | 20q13.12 | 1.06 | 0.0245 | 1 | Over-expressed |
| CHKA | 11q13.2 | 0.62 | 0.0245 | 1 | Over-expressed |
| SYTL4 | Xq22.1 | -0.8 | 0.0246 | 1 | Under-expressed |
| NCF2 | 1q25.3 | -0.64 | 0.0246 | 1 | Under-expressed |
| TFCP2L1 | 2q14.2 | 1.1 | 0.0246 | 1 | Over-expressed |
| HBEGF | 5q31.3 | -0.83 | 0.0246 | 1 | Under-expressed |
| YY2 | Xp22.12 | -0.65 | 0.0247 | 1 | Under-expressed |
| RARB | 3p24.2 | -1 | 0.0247 | 1 | Under-expressed |
| ZNF223 | 19q13.31 | 0.56 | 0.0249 | 1 | Over-expressed |
| SLC25A42 | 19p13.11 | 0.67 | 0.025 | 1 | Over-expressed |
| SYTL3 | 6q25.3 | 0.65 | 0.0251 | 1 | Over-expressed |
| KCTD15 | 19q13.11 | -0.64 | 0.0253 | 1 | Under-expressed |
| FCN1 | 9q34.3 | -0.9 | 0.0263 | 1 | Under-expressed |
| SH3YL1 | 2p25.3 | 0.54 | 0.0267 | 1 | Over-expressed |
| L3HYPDH | 14q23.1 | -0.7 | 0.0269 | 1 | Under-expressed |
| PAX8-AS1 | 2q14.1 | 1.36 | 0.0269 | 1 | Over-expressed |
| CDKN1A | 6p21.2 | -0.81 | 0.0271 | 1 | Under-expressed |
| HPS3 | 3q24 | -0.52 | 0.0271 | 1 | Under-expressed |
| ZFP82 | 19q13.12 | 0.58 | 0.0273 | 1 | Over-expressed |
| IPW | 15q11.2 | 0.61 | 0.0275 | 1 | Over-expressed |
| TNFSF9 | 19p13.3 | -1.14 | 0.0277 | 1 | Under-expressed |
| VSIG4 | Xq12 | -0.95 | 0.0277 | 1 | Under-expressed |
| SCG2 | 2q36.1 | -1.1 | 0.028 | 1 | Under-expressed |
| QPCTL | 19q13.32 | 0.54 | 0.0282 | 1 | Over-expressed |
| MAP3K5 | 6q23.3 | -0.71 | 0.0284 | 1 | Under-expressed |
| OAS2 | 12q24.13 | -0.68 | 0.0284 | 1 | Under-expressed |
| IQGAP2 | 5q13.3 | -0.82 | 0.0284 | 1 | Under-expressed |
| TBX3 | 12q24.21 | -0.75 | 0.0286 | 1 | Under-expressed |
| GRK4 | 4p16.3 | 0.64 | 0.0287 | 1 | Over-expressed |
| ID3 | 1p36.12 | -0.73 | 0.0287 | 1 | Under-expressed |
| NTN4 | 12q22 | -0.89 | 0.0291 | 1 | Under-expressed |
| GPT2 | 16q11.2 | 0.68 | 0.0297 | 1 | Over-expressed |
| ANKRD65 | 1p36.33 | 1.06 | 0.0298 | 1 | Over-expressed |
| RASGEF1B | 4q21.21 | -0.71 | 0.0298 | 1 | Under-expressed |
| GIPC3 | 19p13.3 | -0.72 | 0.03 | 1 | Under-expressed |
| ITGB8 | 7p21.1 | -1.3 | 0.03 | 1 | Under-expressed |
| DYSF | 2p13.2 | -0.93 | 0.0302 | 1 | Under-expressed |
| RND3 | 2q23.3 | -0.72 | 0.0305 | 1 | Under-expressed |
| ADCY4 | 14q12 | -0.61 | 0.0306 | 1 | Under-expressed |
| FOXL1 | 16q24.1 | -0.91 | 0.0307 | 1 | Under-expressed |
| SH3RF2 | 5q32 | -1.89 | 0.031 | 1 | Under-expressed |
| EPB41L3 | 18p11.31 | -0.88 | 0.0311 | 1 | Under-expressed |
| ZNF439 | 19p13.2 | 0.53 | 0.0311 | 1 | Over-expressed |
| ZNF285 | 19q13.31 | 1.31 | 0.0315 | 1 | Over-expressed |
| OSBPL10 | 3p23 | -0.66 | 0.0315 | 1 | Under-expressed |
| LPCAT2 | 16q12.2 | -0.77 | 0.0319 | 1 | Under-expressed |
| NEIL1 | 15q24.2 | 0.58 | 0.0319 | 1 | Over-expressed |
| BAMBI | 10p12.1 | -1.48 | 0.032 | 1 | Under-expressed |
| EFR3B | 2p23.3 | 1.31 | 0.0324 | 1 | Over-expressed |
| IL10 | 1q32.1 | -0.89 | 0.0325 | 1 | Under-expressed |
| FRS3 | 6p21.1 | 0.5 | 0.0326 | 1 | Over-expressed |
| GATA3 | 10p14 | -0.97 | 0.0328 | 1 | Under-expressed |
| TNFRSF21 | 6p12.3 | -0.97 | 0.0331 | 1 | Under-expressed |
| ABLIM3 | 5q32 | -0.77 | 0.0332 | 1 | Under-expressed |
| ADAMTSL4 | 1q21.2 | -0.94 | 0.0337 | 1 | Under-expressed |
| FAAH2 | Xp11.21 | 0.53 | 0.0337 | 1 | Over-expressed |
| IFI44L | 1p31.1 | -0.73 | 0.0338 | 1 | Under-expressed |
| BTBD9 | 6p21.2 | 0.72 | 0.0339 | 1 | Over-expressed |
| SQSTM1 | 5q35.3 | -0.63 | 0.0342 | 1 | Under-expressed |
| EMP1 | 12p13.1 | -0.68 | 0.0343 | 1 | Under-expressed |
| ADGRB2 | 1p35.2 | -0.68 | 0.0343 | 1 | Under-expressed |
| SFTPB | 2p11.2 | 1.71 | 0.0344 | 1 | Over-expressed |
| GNAI1 | 7q21.11 | -0.96 | 0.0346 | 1 | Under-expressed |
| NRXN2 | 11q13.1 | -0.83 | 0.0347 | 1 | Under-expressed |
| ALOX5 | 10q11.21 | -0.56 | 0.0348 | 1 | Under-expressed |
| PRORSD1P | 2p16.1 | 0.56 | 0.035 | 1 | Over-expressed |
| C5AR1 | 19q13.32 | -0.77 | 0.0351 | 1 | Under-expressed |
| PAFAH1B3 | 19q13.2 | 0.69 | 0.0352 | 1 | Over-expressed |
| SLC2A4 | 17p13.1 | -0.75 | 0.0354 | 1 | Under-expressed |
| CCL22 | 16q21 | -1.2 | 0.0355 | 1 | Under-expressed |
| PPP4R1L | 20q13.32 | 0.62 | 0.0357 | 1 | Over-expressed |
| CAV1 | 7q31.2 | -0.74 | 0.0358 | 1 | Under-expressed |
| IFIH1 | 2q24.2 | -0.51 | 0.0359 | 1 | Under-expressed |
| GNAL | 18p11.21 | -1.34 | 0.0359 | 1 | Under-expressed |
| NFE2L3 | 7p15.2 | -0.72 | 0.0362 | 1 | Under-expressed |
| RPS10P7 | 1q32.1 | 0.57 | 0.0367 | 1 | Over-expressed |
| SLC25A45 | 11q13.1 | 0.71 | 0.0367 | 1 | Over-expressed |
| CABLES1 | 18q11.2 | 0.81 | 0.0367 | 1 | Over-expressed |
| COQ2 | 4q21.22-q21.23 | -0.54 | 0.0368 | 1 | Under-expressed |
| OVGP1 | 1p13.2 | 0.84 | 0.037 | 1 | Over-expressed |
| RCAN2 | 6p12.3 | -0.81 | 0.0371 | 1 | Under-expressed |
| PTPRG | 3p14.2 | -0.75 | 0.0372 | 1 | Under-expressed |
| TMEM175 | 4p16.3 | 0.6 | 0.0373 | 1 | Over-expressed |
| GLI2 | 2q14.2 | -0.72 | 0.0375 | 1 | Under-expressed |
| AS3MT | 10q24.32 | 0.68 | 0.0375 | 1 | Over-expressed |
| C1ORF210 | 1p34.2 | 0.59 | 0.0378 | 1 | Over-expressed |
| CRISPLD1 | 8q21.13 | 1.59 | 0.0379 | 1 | Over-expressed |
| SAMD9L | 7q21.2 | -0.58 | 0.0379 | 1 | Under-expressed |
| KLF6 | 10p15.2 | -0.61 | 0.038 | 1 | Under-expressed |
| FAM174B | 15q26.1 | 0.64 | 0.0386 | 1 | Over-expressed |
| FBXO32 | 8q24.13 | -0.77 | 0.0386 | 1 | Under-expressed |
| BCAT2 | 19q13.33 | 0.65 | 0.039 | 1 | Over-expressed |
| TMEM91 | 19q13.2 | 0.77 | 0.0392 | 1 | Over-expressed |
| IL1RN | 2q14.1 | -0.86 | 0.0396 | 1 | Under-expressed |
| FKBP11 | 12q13.12 | 0.59 | 0.0397 | 1 | Over-expressed |
| USP22 | 17p11.2 | 0.5 | 0.0401 | 1 | Over-expressed |
| ST3GAL3 | 1p34.1 | 0.68 | 0.0403 | 1 | Over-expressed |
| CCL2 | 17q12 | -0.67 | 0.0406 | 1 | Under-expressed |
| LIPE | 19q13.2 | 0.72 | 0.0408 | 1 | Over-expressed |
| DCBLD2 | 3q12.1\|3 | -0.66 | 0.041 | 1 | Under-expressed |
| NPR3 | 5p13.3 | -0.96 | 0.0412 | 1 | Under-expressed |
| USP32P1 | 17p11.2 | 0.98 | 0.0414 | 1 | Over-expressed |
| JUNB | 19p13.13 | -0.67 | 0.0414 | 1 | Under-expressed |
| ZNF860 | 3p23-p22.3 | -0.6 | 0.0415 | 1 | Under-expressed |
| NT5E | 6q14.3 | -1.01 | 0.0416 | 1 | Under-expressed |
| NTHL1 | 16p13.3 | 0.55 | 0.0418 | 1 | Over-expressed |
| NCALD | 8q22.3 | 0.83 | 0.042 | 1 | Over-expressed |
| TBKBP1 | 17q21.32 | -0.51 | 0.0421 | 1 | Under-expressed |
| ZNF280B | 22q11.22 | 0.78 | 0.0421 | 1 | Over-expressed |
| ITGA1 | 5q11.2 | -0.71 | 0.0422 | 1 | Under-expressed |
| SDCBP2 | 20p13 | -0.84 | 0.0422 | 1 | Under-expressed |
| DHRS9 | 2q31.1 | -1.21 | 0.0422 | 1 | Under-expressed |
| ZNF222 | 19q13.31 | 0.52 | 0.0425 | 1 | Over-expressed |
| IL3RA | Xp22.33 and Yp11.2 | -0.5 | 0.0426 | 1 | Under-expressed |
| CEP19 | 3q29 | -0.73 | 0.0426 | 1 | Under-expressed |
| NUAK2 | 1q32.1 | 0.62 | 0.0428 | 1 | Over-expressed |
| LSR | 19q13.12 | 0.68 | 0.043 | 1 | Over-expressed |
| CPNE1 | 20q11.22 | 0.72 | 0.0431 | 1 | Over-expressed |
| FN3K | 17q25.3 | 0.5 | 0.0432 | 1 | Over-expressed |
| NQO2 | 6p25.2 | -0.68 | 0.0434 | 1 | Under-expressed |
| FKBP2 | 11q13.1 | 0.69 | 0.0435 | 1 | Over-expressed |
| DYNLT3 | Xp11.4 | -0.51 | 0.0442 | 1 | Under-expressed |
| DCUN1D3 | 16p12.3 | -0.65 | 0.0449 | 1 | Under-expressed |
| CES1 | 16q12.2 | -1.38 | 0.0449 | 1 | Under-expressed |
| FTL | 19q13.33 | -0.53 | 0.045 | 1 | Under-expressed |
| NFYC-AS1 | 1p34.2 | 0.64 | 0.0455 | 1 | Over-expressed |
| FBXO27 | 19q13.2 | -1.54 | 0.0458 | 1 | Under-expressed |
| ATP2C2 | 16q24.1 | 0.96 | 0.046 | 1 | Over-expressed |
| MYADM | 19q13.42 | -0.71 | 0.0462 | 1 | Under-expressed |
| MACROD2 | 20p12.1 | -0.95 | 0.0464 | 1 | Under-expressed |
| PLD1 | 3q26.31 | -0.77 | 0.0467 | 1 | Under-expressed |
| SHANK2 | 11q13.3-q13.4 | 0.73 | 0.0468 | 1 | Over-expressed |
| TMEM200A | 6q23.1 | -0.81 | 0.0468 | 1 | Under-expressed |
| GRIP1 | 12q14.3 | -0.83 | 0.0471 | 1 | Under-expressed |
| AP1S1 | 7q22.1 | -0.54 | 0.0472 | 1 | Under-expressed |
| ACKR4 | 3q22.1 | -0.58 | 0.0473 | 1 | Under-expressed |
| AREG | 4q13.3 | -1.78 | 0.048 | 1 | Under-expressed |
| TXNIP | 1q21.1 | -0.78 | 0.0481 | 1 | Under-expressed |
| P3H2 | 3q28 | -1.28 | 0.0483 | 1 | Under-expressed |
| UBTD1 | 10q24.1-q24.2 | 0.61 | 0.0484 | 1 | Over-expressed |
| SLC35E4 | 22q12.2 | -0.75 | 0.0485 | 1 | Under-expressed |
| B9D2 | 19q13.2 | 0.57 | 0.0486 | 1 | Over-expressed |
| SAMHD1 | 20q11.23 | -0.54 | 0.0487 | 1 | Under-expressed |
| IL18R1 | 2q12.1 | -0.75 | 0.0488 | 1 | Under-expressed |
| ZNF284 | 19q13.31 | 0.65 | 0.0491 | 1 | Over-expressed |
| ZNF33B | 10q11.21 | 0.5 | 0.0492 | 1 | Over-expressed |
| PPARG | 3p25.2 | -1.15 | 0.0492 | 1 | Under-expressed |
| MCTP1 | 5q15 | -1.15 | 0.0493 | 1 | Under-expressed |
