# Supplementary material for: Function of low ADARB1 expression in lung adenocarcinoma
Source: PLoS One. 2019 Sep 6;14(9):e0222298. doi: 10.1371/journal.pone.0222298 (PMC6730894; doi:10.1371/journal.pone.0222298)
Supplement: S4 Table — (DOCX) [file pone.0222298.s004.docx]

Supplemental table 4 KEGG pathway of ADARB1-associated co-DEGs in LUAD patients.

| **Term** | **Count** | **P Value** | **Genes** |
| --- | --- | --- | --- |
| hsa01210:2-Oxocarboxylic acid metabolism | 3 | 0.033 | BCAT2, IDH1, GPT2 |
| hsa05150:Staphylococcus aureus infection | 4 | 0.064 | C5AR1, CFH, ITGAM, IL10 |
| hsa04068:FoxO signaling pathway | 6 | 0.078 | SGK1, CDKN1A, SLC2A4, FBXO32, GADD45B, IL10 |
| hsa04915:Estrogen signaling pathway | 5 | 0.088 | ADCY4, HSPA2, GNAI1, FKBP4, HBEGF |
| hsa04062:Chemokine signaling pathway | 7 | 0.097 | ADCY4, CCL22, CCL2, TIAM1, GNAI1, GRK4, PXN |
